# An Improved Population Migration Algorithm Introducing the Local Search Mechanism of the Leap-Frog Algorithm and Crossover Operator

**DOI:** 10.1371/journal.pone.0056652

**Published:** 2013-02-27

**Authors:** Yanqing Zhang, Xueying Liu

**Affiliations:** Department of Mathematics, Inner Mongolia University of Technology, Hohhot, China; Dana-Farber Cancer Institute, United States of America

## Abstract

The population migration algorithm (PMA) is a simulation of a population of the intelligent algorithm. Given the prematurity and low precision of PMA, this paper introduces a local search mechanism of the leap-frog algorithm and crossover operator to improve the PMA search speed and global convergence properties. The typical test function verifies the improved algorithm through its performance. Compared with the improved population migration and other intelligential algorithms, the result shows that the convergence rate of the improved PMA is very high and its convergence is proved.

## Introduction

The population migration algorithm (PMA) was proposed by Zhou et al in 2003 [1]–[2]. The PMA is a simulated population migration theory global optimization algorithm. The PMA is also a simulated mechanism that involves population along with economic center transfer and population pressure diffusion in the field. In other words, people relocate to a preferential region that has a high level of economic development and employment opportunities. When the preferential region has relative overpopulation, the population pressure constantly increases. When population pressure exceeds a certain limit, people will move to a more suitable preferential region. Population pressure makes the PMA engage in a better regional search. To a certain extent, population proliferation can prevent the PMA from falling into a local optimal solution. The whole algorithm shows alternate features that localized search and scatter search have in the search process.

In 2004, Xu gave an improved PMA (IPMA), which can be summarized into three forms: population flow, population migration, and population proliferation [3]. Population flow states that the population residing in the region is spontaneous and the overall flow is uncertain. Population migration is based on the mechanism across a wide range of the selection movement, in which people flow to the rich place. When the population pressure is very high, population proliferation is a selective movement from a preferential area to a no preferential area. It also reflects the people’s pioneering spirit.

Recent, various kinds of intelligent algorithms have been improved by many researchers. Wang et al improved the PMA with the steepest descent operator and the Gauss mutation [4]. Ouyang et al introduced the simplex in the PMA to improve the search performance of the algorithm [5]. Guo et al increased performance of the population migration by improving the migrations’ efficiency [6]. Karaboga improved the bee swarm [7]. Li et al introduced extremal optimization to improve the performance of the shuffled frog leaping algorithm (SFLA) [8]. However, the results of all these studies show low accuracy or fall into the local optimal solution. To further improve the local search ability of the PMA and speed up convergence, we introduce a local search mechanism of frog leaping and crossover. This paper is improved and uses a number of classic functions for the simulation to show that the algorithm is effective and feasible.

### Population Migration Algorithm

Consider the following unconstrained single-objective optimization problem:
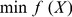
(1)

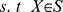



In which 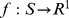 is a real valued mapping, 
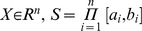
 is a search space, 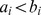. We assume that problem (1) has a constant solution, namely, global optimal solution exists and the set *H* of the global optimal solution is not empty. Seeking the most value problem can be transformed into the form of problem (1). In the algorithm, 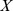 is an individual and stands for the population and various kinds of information. 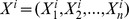 is the *ith* individual in the solution space, 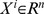; 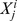
is the *ith* individual of the *jth* weight; 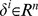, 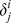 is the *jth* weight of the *ith* region’s radius 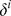, in which, 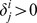, 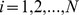, 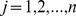; and *N* stands for population size. Target function *f*(*x*) corresponds to the population attraction. The optimal solution (a local optimal solution) of problem (1) corresponds to the most attractive region (the preferential). The ability of the PMA to escape from the local optimal solution corresponds to the ability. Due to population pressure increase, the population moves out of the preferential area. The rise of algorithm or mountain climbing made the populations move into the preferential area. Local random search of algorithm corresponds to the population flow. The method by which the algorithm selects the approximate solution corresponds to the population migration. Escaping from the local optimal strategies corresponds to the population proliferation.

### Frog Leaping Algorithm of Local Search

SFLA is proposed, as proved by Eusuff in 2003 [9]. SFLA is based on the heuristic and collaborative search of species. The implementation of the algorithm simulates the element evolution behavior of nature.

The implementation process of SFLA simulates the feeding behavior of a group of frogs in a wetland [10]. In the feeding process, every frog can be seen as the carrier of ideas and information. Frogs can communicate through the exchange of information, which can improve the knowledge of others. Every frog represents a solution to the optimization problem (1). Wetland represents the solution space. In the algorithm implemental stage, there are *F* frogs divided into *N* groups. Each group has *l* frogs. Different groups have different ideas and information. In a group, frogs follow the element evolution strategy in the solution space for local depth search and internal communication.
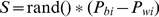
(2)

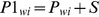
(3)


In the above formulas, rand() is a random function that randomly generates the value in the range [0,1], *S* is a step of the frog leaping, *P_wi_* stands for the worst frog of the *ith* group, *P_bi_* stands for the best frog of the *ith* group, and *P*1*_wi_* stands for an update of the worst frog of the *ith* group. If the adaptive value of the updated worst frog is more than that of the original worst frog in the same group, the updated worst frog will replace the original worst frog. Otherwise, a frog will be randomly generated in the group instead of the original worst frog. The updated process is repeated until the predetermined number of local search *L_S_*. When all groups have completed the depth of local search, all the frogs of the groups are again mixed and divided into *N* groups. We continue the local search in the groups until the termination rule holds true, and then we stop.

### Crossover Operators

By two leaner combinations, we can obtain two new individuals. Arithmetic crossover operation is often used to express individuals by floating individual coding. Hypothesis: The two parents are 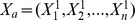 and 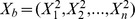.

Then, *n* random numbers are randomly generated, in which 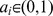, 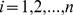. After hybrid implementation, two new progenies are generated:
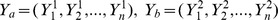


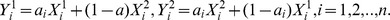



## Methods

In the PMA, the population flow includes extensive information exchange. In practice, population information exchange decides the direction of the migration under certain conditions, so we should give full attention to population flow. When under pressure, population migration is the random population flow in the range of the population pressure, which does not have a significant role in further improving the algorithm.

The local search mechanism of frog-leaping algorithm and the crossover operator are introduced into the PMA in the process of population flow, which can well improve the original search results. IMPA organically made the frog-leaping combine with the crossover operator in the PMA. The process of the algorithm is as follows:

### Initialization

Input the population size *N*, the initial radius *δ*(0), the vigilance parameter of population pressure *α*(0), the number of the population flow *l*, the number of local search *L_S_*, and the maximum number of iterations *T*. We randomly generate *N* individuals in the search space *S*, *X* ^1^, *X* ^2^, …, *X^N^*. The *ith* regional center 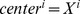 determines the upper and lower 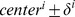
bounds of the *ith* region, in which , 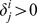, 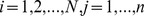.The extraction method 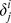 makes 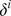 equal, so we cancel the superscript of 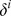 in the following steps. We obtain an initial search space 
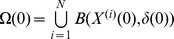
, which stands for the initial residential area. The set 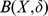 is taken as the sphere, in which *X* is the center and *r* is the radium. The following variables appearing that way are the same. 
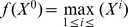
 and 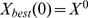. We set iteration counter *t* = 0.

### Evolutionary

1. Preparation: 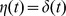

2. Population flow: In every 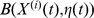, we randomly generate *l_i_* individuals, so we can obtain the group 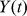 in 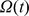, which has *Nl* individuals.
3. Population migration
  We next introduce the local search mechanism of frog-leaping algorithm and crossover operator. At this time, the role of population has been transformed to the frog. The search area is seen as the frog’s wetland. The formed *N* local areas are seen as the *N* groups. The individual of every local area is seen as the frog.
  (a) We choose *N* best frogs in *Y*(*t*), which form the intermediate frog group 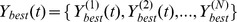.
  (b) Forming *N* regions: 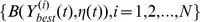.
  (c) In every group, we produce a frog body flow. In other words, *l_i_* individuals are randomly generated in 
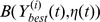
. *l_i_* and the adaptive value of 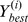
are proportional.
  (d) We set the group counter *iN* = 1, which is compared with the total of groups *N*; the local evolution counter *im* = 1, which is compared with the number of local search *L_S_*.
  (e) In the *iNth* group, we calculate the adaptive value of every frog in the group and arrange them in descending order. We can obtain the best solution *P_bi_* and the worst solution *P_wi_*.
  (f) *Ph_wi_* = *P_wi_*, according to the following four formulas, we can update and improve the position of the worst frog in the group.

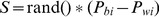
(4)

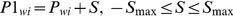
(5)

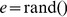
(6)

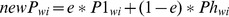
(7)
  (g) If step (f) improved the position of the worst frog, the adaptive value of 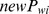
is more than the *P_wi_*’s, the position of *P_wi_* will be replaced by the position of 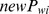.That is, 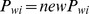. Otherwise, we randomly generate a position of a frog instead of the worst frog in the group.
  (h) If *im*<*L_S_*, then *im* = *im*+1and turn to step (f). Otherwise, we report the best frog 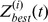 and turn to step (i).
  (i) If *iN*<*N*, then *iN* = *iN+*1 and we turn to step (e). Otherwise, we turn to step (j).
  (j) Contraction of preferential region: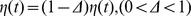.
  (k) If 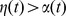 (not more than the population pressure alert), we continue the frog flow and turn to step (c) Otherwise, we turn to step (l).
  (l) We report *N* the best frogs: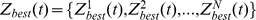.
4. Population proliferation:
  (a) We keep the best individual in the 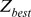, which is credited as 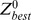.
  (b) In the 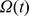, we randomly sample *N*-1 new individuals instead of the *N*-1 individuals of 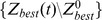.
  (c) We define a new generation of population

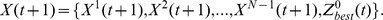



### Termination of Inspection

If the new generation of population *X*(*t*+1) contains the eligible solution, we stop and output the best solution in *X*(*t*+1). Otherwise, we narrow 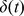 and 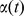 properly and set *t*:*t*+1. If the maximum numbers of iterations for the algorithm are not met, we turn to the second step.

### Convergence Analysis

This paper uses the axiom model to analyze and prove the convergence of IMPA.

**Definition 1** Optimization variables *X* have limited-length characters and have the form 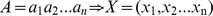. *n* is called the code string length of *X*, *A* is called the code of *X*, and *X* is called the decode of the character *A*. *a_i_* is considered a genetic gene. All possible values of *a_i_* are called allelic gene. *A* is a chromosome made up of *n* genes [11].

**Definition 2** (Individual Space) 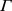
is an allelic gene. *n* is given the code string length. The set 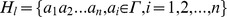 is called individual, in which 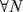 is a natural number and 
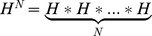
 is the 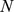
order of population space [11].

**Definition 3** (Satisfaction Set) If 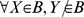, then 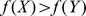. *B*(*X*) is called the satisfaction set. The number of individuals in the set *B*(*X*) is used for 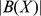. 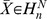 is any of the population and  is the best individual in the set . The satisfaction set induced by 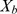
is called satisfaction set of the population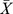, which is marked 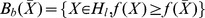. All of the satisfaction sets’ intersections are formed with the global optimal solution 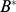 [11].

**Definition 4** (Selection Operator) The selection operator 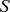
is a random mapping, 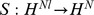. It meets the following two conditions. (1) For 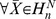, we can obtain 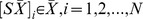, in which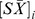 stands for the 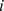
individual in the 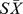; (2) Any population 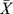
that meets 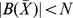
has 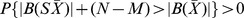. The first condition states that selected individuals should be in the selected population. The second condition states that the selection operator may increase the number of optimal individuals in the population, in addition to variations of the individuals’ numbers between selected population and original population [11].

**Definition 5** (Reproduction Operator) Reproduction operator 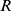
is a random mapping.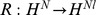. If any population 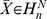, which meets 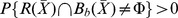, the new population 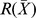 which 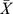
becomes under the action of the operator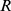, may contain more satisfied individuals than the original population [11].

**Definition 6** (Mutation Operator) Mutation operator 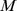
is a random mapping: 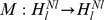.
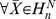
meets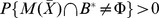. Mutation operator is the reproduction operator that has a much better nature. If the population contains satisfied individuals, then after mutation, the population should contain satisfied individuals. If the population does not have satisfied individuals, then after mutation, the population may contain satisfied individuals [11].

In improved population migration, extended operator 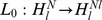 meets the following expressions:
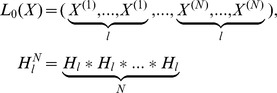
and



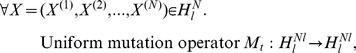


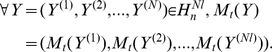



Therefore, we can use the operator 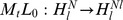 to stand for the process of population flow. The selection operator *S*_1_ stands for the process of step (a) in population migration, where we chose *N*, the best individuals from population *Y*(*t*). The reproduction operator *R*_1_ stands for the progress from step (b) to the process of final output *Z_best_*(*t*) in step (l). The reproduction operator *R*_2_stands for the process from population proliferation to finally generate *X*(*t*+1). Therefore, the IPMA can be expressed as:
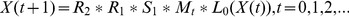
(8)

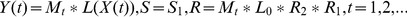
(9)


After both sides have the same function of operator 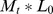 for (8), we can find that 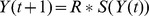.The general simulated evolution algorithm can be composed of a series of selection operators 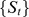 and reproduction operators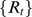 to be represented abstractly as follows: 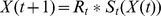.

**Lemma 1** In the IPMA,*S* is a selection operator and *R* is a reproduction operator. The proof can be obtained from reference [11].

**Theorem 1** The IPMA is a simulated evolutionary algorithm.

**Lemma 2** In the IPMA, the characteristic number of the preferred selection operator, the selection pressure of the operator *S*:*P_S_* = 1, the selection intensity *α_S_* = 1. The IPMA just takes the preferred selection operator. According to reference [3], the selection pressure and the selection intensity are defined to calculate the characteristic number of the operator *S*. The proof can be obtained from reference [11].

**Lemma 3** In the IPMA, the characteristic number of the reproduction operator, the operator 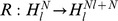, then the absorption and scattering rate of the reproduction meet the following estimates: 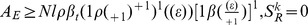. Where, 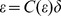
(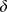
is the radius of the search space) is the radius of the set of optimal solution 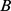 and 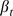 is a conversion coefficient. If the set 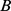
includes a division region in the 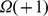, then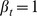; otherwise, 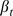
is the rate of volume of the intersection of the set 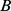
and a division region. Thus, we can obtain 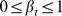. The proof can be obtained from reference [11].

**Lemma 4** If the abstract simulated evolutionary algorithm (SEA) satisfies the following conditions: (1) the pressure of selection operator 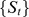 is uniformly bounded; (2) the absorption of reproduction operator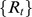: 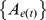 meets 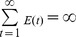; (3) the strength of selection 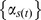, absorption 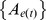, and scattering 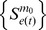
content are as follows: 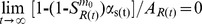. Then, an abstract and a general SEA will probably weakly approach the optimal solution set. The proof can be obtained from reference [11].

**Theorem 2** (Convergence of IPMA) If the IPMA adopts the selection operator family 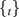, which comprise all the preferred selection operators, reproduction operators family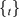, in which 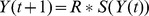. The following conditions are thus met: (1); (2)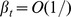 (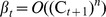).

Then, to prove that the IPMA will probably weakly approach the solution set, the following steps are performed.

#### Proving

Known by lemma 2: 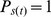,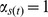; Known by lemma 3: 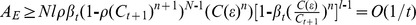,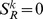; when 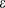 is given, 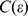
is also given. By condition (1), we obtain 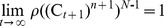. By condition (2), we obtain 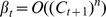, then 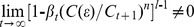. We have 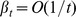 and lemma 3, then 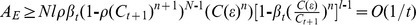. We know the divergence of the harmonic series, so 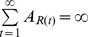. According to lemma 3: 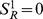. According to lemma 2: 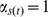, so 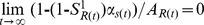. The three conditions of lemma 3 are met. The IPMA probably weakly approaches the global optimal solution.

## Results and Discussion

### Simulation Examples and Experimental Parameter Setting

To verify the performance of the IPMA, this paper performs an experiment. The IPMA is compared with the basic population algorithm and other improved algorithms. In the simulation experiment, we choose 10 classic test functions to test the algorithm. According to the performance of the functions, they are divided into a single minimum (single) and a number of local minima (multimodal) into two categories. The following lists the 10 functions’ definition, variable range, and global optimal values of the theory and optimal solution of the theory. The function *f*_1_ is a unimodal function used to examine the accuracy of the algorithm. The function *f*_2_ which contains infinite local optimal solution and has infinite local maximum near suboptimal solution, is a strong shock multimodal function. General optimization algorithms easily fall into local optimal solution. Thus, the ability of the algorithm is used to avoid falling into a local optimization algorithm. The function *f*_5_ is a multimodal function. It has about 10*D (D is dimension) local optimal solution. The function *f*_6_ is a deep and local minimum multimodal function that has many local optimal solutions. The functions *f*_5_ and *f*_7_ are complex nonlinears used to test global search performance of algorithms. The function *f*_8_ is a complex and classical function used to test the optimization efficiency of algorithms. In a certain number of iterations, this paper estimates the performance of IMPM by the test function of the best value, the worst value, average value, variance, average running time, and convergence rate.

1. Sphere Model function: 
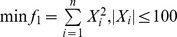
. The optimal solution is 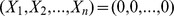 and the optimal minimum value is 0, when *n* = 30.

2. Schaffer function: 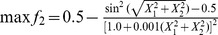, 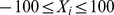, *i* = 1,2. The optimal solution is (*X*_1_, *X*_2_) = (0, 0) and the optimal maximum value is 1.

3. 
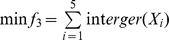
, 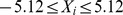, 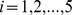. The function in the section (−5.12, −5) has a global minimum value −30.

4. 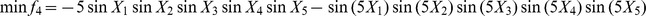, 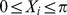, 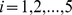. The optimal solution is 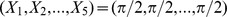
and the optimal minimum value is −6.

5. Generalized Rastrigin function: . The function has many local minimum solutions, but it has only one global minimum solution (*X*_1_, *X*_2_) = (0, 0) and the global minimum value is 0, when *n* = 2, *n* = 5 and *n* = 10.

6. Quartic function: 
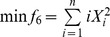
,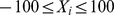, 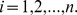. The optimal solution is 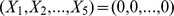 and the optimal minimum value is 0, when *n* = 20.

7. Ackley’s function: 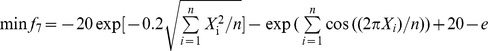, 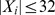. The optimal solution is 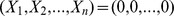 and the optimal minimum value is 0, when *n* = 20.

8. Generalized Rosen Brock’s function:


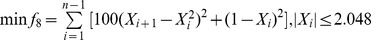
. The optimal solution is 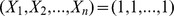 and the optimal minimum value is 0, when *n* = 3.

9. Griewank function: 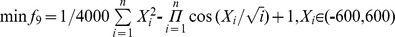. The optimal minimum value is 0, when *n* = 30.

10. Schwefel function: 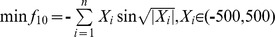. The optimal minimum value is −2094.9, when *n* = 5. The optimal minimum value is −20949, when *n* = 50.

To verify the performance of the IPMA, we choose a computer whose CPU is made by AMD and has an operating system of Windows 7. The mathematical software Matlab7.0 was used for the testing. Each function is applied 50 times. We present the parameters in the process of solving the functions for this paper in the table 1. To show the better performance of the IPMA, we compare it with other algorithms through the following indicators: best and worst solutions, average solution, variance, average running time, and average convergence rate in 50 cycles.

**Table 1:** Parameter setting of the functions for the IPMA.

Function	*N*	*δ*	*l*	*L_S_*	*Δ*	*α*	*K*
1	3	10	10	5	0.1	1E−6	30
2	10	10	10	5	0.1	1E−7	30
3	3	2	10	5	0.1	1E−7	30
4	3	0.5	10	5	0.1	1E−9	30
5	3	1.6	10	5	0.15	1E−8	30
	10	0.512	10	5	0.1	1E−9	30
	10	0.206	10	5	0.1	1E−9	30
6	5	0.15	10	5	0.15	1E−9	30
7	5	5	10	5	0.1	1E−10	30
8	10	0.2	10	5	0.01	1E−9	30
9	10	60	10	5	0.1	1E−9	30
10	10	50	10	5	0.1	1E−6	30

This table shows parameter setting of the ten functions. Population size *N*, number of population flow *l*, radius *δ*, coefficient of contraction *Δ*, pressure of population parameters *α*, number of local search *L_S_*, and largest number of iterations *K*. 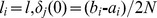.

### Experimental Result and Analysis

In Table 2, the data of function *f*_1_ show that the improved population algorithm is better than frog-leaping algorithm in terms of precision. Other algorithms find it very difficult to achieve the optimal solution of function *f*_2_, but the IPMA can easily do so, which indicates that the algorithm’s ability of escaping from local optimal solution is very ideal. Function *f*_8_ can test whether the algorithm’s ability of global optimization is good. It is used to measure the efficiency of the algorithm, so we know the efficiency of the IPMA is very high compared with other algorithms from the data of function *f*_8_.The data functions *f*_3_, *f*_4_, *f*_5_, *f*_7_, *f*_9_ and *f*_10_ reflect that the IPMA is a better algorithm from a certain angle. Through cooperation of the best value, the worst value, the average value, the variance, and the convergence rate, the IPMA is found to be better than the other algorithms in the calculation of stability and precision.

**Table 2:** Comparison performance of the IPMA in the ten functions.

Function	Algorithm	Best value	Worst value	Average value	Variance	Average running time	Convergence rate
1	[12]	_10_^−13^	_10_^−6^	_10_^−7^	_10_^−7^	26.8	–
	[6]	1.52E−02	5.47E−02	_10_^−2^	–	–	–
	IPMA	_10_^−18^	_10_^−12^	_10_^−13^	_10_^−25^	0.749	100%
2	[1]	1.000000	0.990284	–	–	–	75%
	[4]	1.000000	1.000000	–	–	4.768	–
	[5]	1E−10	1E−10	–	–	7.626	–
	IPMA	1	1	1	0	2.104	100%
3	[1]	−30	−25	–	–	–	75%
	[4]	−30	−29	–	–	2.881	95%
	IPMA	−30	−29	29.86	0.2040	0.542	96%
4	[4]	−6.000000	−6.000000	−	–	7.015	100%
	IPMA	−6	−6	−6	0	0.704	100%
5	[4]	0	0	0	0	4.047	100%
	[7]	–	–	4.68E−17	2.64E−17	–	–
	IPMA	0	0	0	0	0.614	100%
	[8]	–	–	0	–	0.27	–
	IPMA	0	0	0	0	1.529	100%
	[6]	0	–	–	–	–	40%
	IPMA	0	0	0	0	2.731	100%
6	[4]	_10_^−17^	_10_^−17^	–	–	23.375	100%
	IPMA	_10_^−21^	_10_^−18^	_10_^−19^	_10_^−35^	1.043	100%
7	[12]	3.693E−07	2.738	6.375E−01	7.788E−01	29	–
	[4]	_10_^−10^	_10_^−10^	–	–	29.893	100%
	[6]	3.59E−02	9.94E−02	10^−2^	–	–	–
	[8]	–	–	_10_^−2^	–	0.42	–
	IPMA	_10_^−13^	_10_^−11^	_10_^−11^	_10_^−21^	1.675	100%
8	[1]	_10_^−6^	_10_^−6^	–	–	–	100%
	[12]	4.667	2.920E+02	6.690E+01	5.512E+01	50.0	–
	[7]	–	–	0.002234	0.002645	–	–
	IPMA	_10_^−19^	_10_^−17^	_10_^−18^	_10_^−36^	10.013	100%
9	[8]	–	–	_10_^−2^	–	0.21	–
	IPMA	0	0	0	0	3.52	100%
10	[8]	–	–	−2094.57	–	0.06	–
	IPMA	−2094.90	−2094.90	−2094.90	0	11.12	100%
	[8]	–	–	−20946.58	–	1.97	–
	IPMA	−20949.00	–20949.00	−20949.00	0	5.99	100%

This table shows performance of IPMA compared with other algorithms through the above indicators in 50 cycles.

### Conclusions

In this paper, based on the local search mechanism and crossover operator, we put forward an IPMA. The parameters of the IPMA are simple and easy to realize. By testing the 10 functions, we find that the IPMA is better than the basic PMA and is superior to many intelligent algorithms. The PMA is good at global searches, but it does not perform well in local search and its precision is low. We introduce local search in population and crossover operator to improve these deficits. Therefore, the IPMA is increased to avoid falling into local optimum capacity; its calculation speed and precision are also improved.
